# Lung transplantation for pulmonary fibrosis in dyskeratosis congenita: Case Report and systematic literature review

**DOI:** 10.1186/1471-2326-11-3

**Published:** 2011-06-15

**Authors:** Neelam Giri, Rees Lee, Albert Faro, Charles B Huddleston, Frances V White, Blanche P Alter, Sharon A Savage

**Affiliations:** 1 Division of Cancer Epidemiology and Genetics, Clinical Genetics Branch, National Cancer Institute, National Institutes of Health, Rockville 20852, MD, USA; 2 Department of Pediatrics, Pediatric Pulmonary Medicine, Naval Medical Center, Portsmouth, VA 23708, USA; 3 Department of Pediatrics, Division of Pulmonary Medicine, Washington University School of Medicine, St. Louis, MO 63110, USA; 4 Department of Surgery, Washington University School of Medicine, St. Louis, MO 63110, USA; 5 Department of Pathology and Immunology, Washington University School of Medicine, St. Louis, MO 63110, USA

## Abstract

**Background:**

Dyskeratosis congenita (DC) is a progressive, multi-system, inherited disorder of telomere biology with high risks of morbidity and mortality from bone marrow failure, hematologic malignancy, solid tumors and pulmonary fibrosis. Hematopoietic stem cell transplantation (HSCT) can cure the bone marrow failure, but it does not eliminate the risks of other complications, for which life-long surveillance is required. Pulmonary fibrosis is a progressive and lethal complication of DC.

**Case presentation:**

In this report, we describe a patient with DC who developed pulmonary fibrosis seven years after HSCT for severe aplastic anemia, and was successfully treated with bilateral lung transplantation. We also performed a systematic literature review to understand the burden of pulmonary disease in patients with DC who did or did not receive an HSCT. Including our patient, we identified 49 DC patients with pulmonary disease (12 after HSCT and 37 without HSCT), and 509 with no reported pulmonary complications.

**Conclusion:**

Our current case and literature review indicate that pulmonary morbidity is one of the major contributors to poor quality of life and reduced long-term survival in DC. We suggest that lung transplantation be considered for patients with DC who develop pulmonary fibrosis with no concurrent evidence of multi-organ failure.

## Background

Dyskeratosis congenita (DC) is a progressive, multi-system, inherited disorder of telomere biology. It is classically diagnosed by the presence of the triad of nail dystrophy, lacy reticular pigmentation, and oral leukoplakia. Patients with DC are at very high risk of bone marrow failure (BMF), squamous cell head and neck or other cancers, leukemia, and myelodysplastic syndrome (MDS), as well as pulmonary fibrosis, liver disease, neurological, ophthalmic, genitourinary, and gastrointestinal abnormalities [1,2].

Telomeres, which consist of TTAGGG nucleotide repeats and a protein complex at chromosome ends, are essential for chromosome stability. They are generally very short in individuals with DC [3]. Approximately 60% of persons with DC have an identifiable mutation in one of seven genes important in telomere biology. Inheritance of DC may follow X-linked recessive (*DKC1 *gene), autosomal dominant (*TERC, TERT*, or *TINF2)*, or autosomal recessive patterns (*NOP10, NHP2, TERT*, or *TCAB1*) [4-10]. BMF is the leading cause of death, accounting for 60-70% of all fatalities [2,11,12]. Hematopoietic stem cell transplantation (HSCT) can correct BMF and other hematologic complications (*i.e.*, MDS or leukemia), but it does not improve other DC-related manifestations.

Pulmonary fibrosis is a progressive and lethal complication of DC, even in the absence of BMF, accounting for more than 15% of deaths in patients with DC [11]. Idiopathic pulmonary fibrosis (IPF), which is pathologically similar to DC-related pulmonary fibrosis, is a diffuse parenchymal lung disease of unknown origin, reported mainly in adults [13]. Up to 10% of IPF patients have a germline mutation in either telomerase (*TERT) *or its RNA component (*TERC*) despite lacking other signs or symptoms of DC [14,15], implying that these two conditions are part of a broad spectrum of telomere biology disorders. IPF can be effectively treated with lung transplantation [16]. Lung transplantation is also an accepted therapy for children with end-stage pulmonary disease for conditions such as cystic fibrosis, idiopathic pulmonary hypertension, congenital heart disease, post allogeneic HSCT-related lung damage, and interstitial lung disease [17,18]. However, to our knowledge, lung transplantation for DC-related pulmonary fibrosis has not been reported.

Here we describe the first successful bilateral lung transplant in a DC patient who developed pulmonary fibrosis seven years after HSCT for severe aplastic anemia (SAA). We also present a systematic review of the literature on pulmonary disease in patients with DC who did or did not receive a HSCT.

## Case Presentation

The reported patient is enrolled in National Cancer Institute protocol 02-C-0052 (NCT00056121, http://www.marrowfailure.cancer.gov), which is approved by the Institutional Review Board.

Case: A 14-year-old Asian boy (NCI-204-1) presented with anemia and thrombocytopenia at three years of age. This rapidly progressed to SAA, and he underwent a Human Leukocyte Antigen-identical sibling donor HSCT at age four. The preparative regimen consisted of cyclophosphamide (50 mg/kg × 4 days) and anti-thymocyte globulin (30 mg/kg × 3 days). Cyclosporine and methotrexate were used for graft *versus *host disease (GvHD) prophylaxis. He engrafted rapidly, without evidence of acute GvHD, infections or other HSCT-related complications. Review of his medical records indicated that he had a "geographic tongue" and toenail dystrophy noted prior to HSCT, but the diagnosis of DC was not made at that time. During the next two to three years after HSCT, his mucocutaneous findings evolved into the classic DC triad of oral (tongue) leukoplakia, finger and toe nail dystrophy, and reticular skin pigmentation. He also developed bilateral lacrimal duct obstruction, trichiasis and urethral stenosis. This constellation of findings led to the clinical diagnosis of DC at age seven, four years after he presented with BMF. He was subsequently found to have an heterozygous mutation in *TINF2 *(c.851_852delCA resulting in Thr284SerfsX5) in his skin fibroblasts. This mutation results in a truncation of the protein product of *TINF2 *in the same region as other mutations in this gene reported to cause DC [9]. There was no family history of DC-related illnesses, and all family members were negative for mutations in *TINF2*.

**Figure 1:**
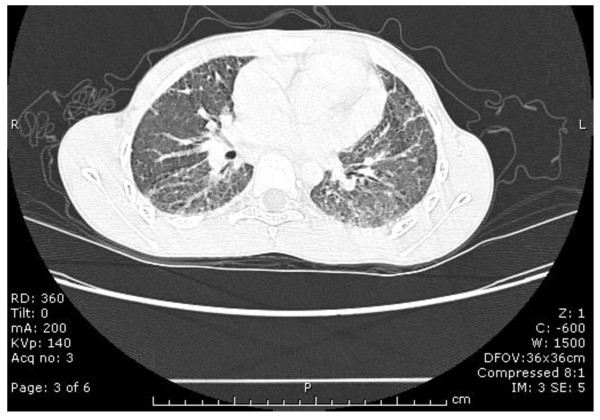
**Chest computerized tomography of the patient at age 11 years, 7 years after hematopoietic stem cell transplantation and 17 months prior to lung transplantation**. At the time of this evaluation, he had dyspnea on exertion, a chronic, non-productive cough, and a restrictive pattern on pulmonary function tests. Bilateral, diffuse areas of ground glass opacities and fibrosis of the lung parenchyma are shown.

At age 11, seven years after HSCT, the patient developed a chronic persistent dry cough and exertional dyspnea. Spirometry revealed a forced vital capacity (FVC) of 0.78L (31% of predicted), forced expiratory volume in 1 second (FEV1) of 0.73L (33% of predicted), and the flow/volume loop suggested a restrictive pattern (Table 1). A short course of oral prednisone (60 mg [2 mg/kg] for 10 days) resulted in transient improvement (FVC 51% predicted; FEV1 48% predicted). Pulmonary function testing (PFT) obtained following the steroid pulse confirmed moderate to severe restrictive lung disease, with total lung capacity (TLC) 56% predicted, mild air trapping (RV/TLC 33%), and a significant reduction in diffusion capacity for carbon monoxide (DLCO) at 27% of predicted (Table 1). Chest radiograph demonstrated a diffuse interstitial pattern which was consistent with pulmonary fibrosis. High-resolution computerized tomography (CT) of the lungs showed bilateral diffuse areas of ground glass opacities and fibrosis (Figure 1). Lung biopsy was interpreted as severe interstitial fibrosis, confirming the clinical diagnosis.

**Table 1:** Pulmonary function at diagnosis of pulmonary fibrosis, before and after lung transplantation

Age in years	11 6/12	11 9/12	12 9/12	14 6/12
Pulmonary function tests	At presentation	After steroid burst	3 months prior to lung transplantation	18 months after lung transplantation

FVC (L), (% predicted)	0.78; (31%)	(51%)	0.51; (20%)	2.29; (81%)

FEV1 (L), (% predicted)	0.73; (33%)	(48%)	0.51, (24%)	1.91; (78%)

FEV1/FVC%	0.94; (109%)	-	100%	83%

DLCO (corrected)	-	27%	-	85%

TLC (L)	-	56%)	1.45; (41%)	2.36; (81%)

FEF 25-75% (L/second)	0.88; (35%)		1.29; (52%)	2.08; (81%)

RV/TLC (% predicted)	-	33%	53%	-

PEFR; (% predicted)	2.86; (61%)	-	1.74; (36%)	-

VQ scan	-	-	Moderate or large areas of perfusion defect	Normal

Lung CT	Interstitial fibrosis	-	Diffuse fibrosis and ground glass opacities. Subpleural reticulation. Bilateral traction bronchiectasis.	Mild atelectasis

Lung biopsy	Pulmonary fibrosis	-	-	No rejection

Abbreviations: CT, computerized tomography; DLCO, diffusion capacity for carbon monoxide; FEF, forced expiratory flow; FEV1, forced expiratory volume in 1 second; FVC, forced vital capacity; HSCT, hematopoietic stem cell transplant; PEFR, peak expiratory flow rate; RV, residual volume; TLC, total lung capacity; VQ scan, pulmonary ventilation/perfusion scan.

The patient received every other day oral steroids (0.5 mg/kg/dose) as maintenance therapy. However, after approximately 9 months, his symptoms progressed and his pulmonary function declined, despite escalating immunosuppressive therapy which included high-dose pulse steroids (30 mg/kg methylprednisolone IV for 3 days per month), alternate day low-dose prednisone (0.5 mg/kg/dose), azathioprine (50 mg daily) and N-acetyl-cysteine (600 mg three times daily). He became wheelchair-bound and required 24-hour ventilatory support with nasal Bi-level Positive Airway Pressure, at age 12. After three months on the lung transplant waiting list a deceased donor was identified. He underwent bilateral lung transplantation at age 13.

**Figure 2:**
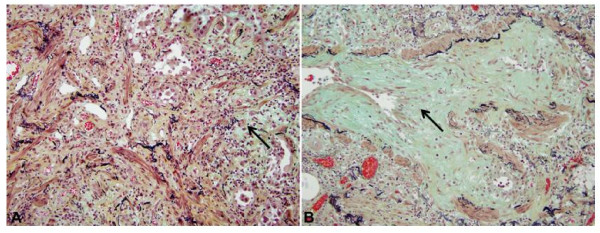
**Pulmonary histology at the time of lung transplantation**. **A**: Representative microscopic field of explanted lung shows diffuse fibrosis, with both established collagen deposition (yellow-green hue) and fibroblastic proliferation (arrow, blue-green hue). There is prominent extension of smooth muscle into interstitium. Residual alveoli are lined by hyperplastic pneumocytes (200x, pentachrome stain). **B**: Bronchiolitis obliterans involving small airway shown by arrow (400x, pentachrome stain).

On gross examination, the explanted lungs were firm and poorly aerated, with thickening of small airway walls. Microscopic examination was consistent with a severe interstitial fibrosing process. Throughout most of the lung, there were both established fibrosis and ongoing fibroblastic proliferation, with obliteration of alveolar spaces, extension of smooth muscle into interstitium and chronic inflammation, and marked pneumocyte hyperplasia of residual alveoli. In addition, there was extensive bronchiolitis obliterans, including both established dense collagen deposition and fibroblastic proliferation within the airways (Figure 2). Alveolar architecture was more intact in a minority of lobules, with mild alveolar septal widening, pneumocyte hyperplasia and fibroblastic proliferation, but without the heterogeneous temporal and peripheral pattern characteristic of usual interstitial fibrosis. Hyaline membranes and end-stage "honey-comb" parenchyma were not present.

The patient is now twenty-one months post-lung transplant, and is doing well, with resolution of respiratory symptoms and an excellent level of activity. He has no evidence of rejection either clinically or on transbronchial biopsy. His immunosuppression consists of tacrolimus, with goal trough levels of 8 ng/ml, and prednisone 12.5 mg daily. Because of mild neutropenia, he does not receive mycophenolate mofetil. His white blood cell count 18 months after lung transplant was 3000/mm^3 ^(normal range 3300 - 8700/mm^3^), with an absolute neutrophil count of 1200/mm^3 ^(normal range 1500 - 6090/mm^3^). His most recent oxyhemoglobin saturation was 99-100% on room air; and FVC, FEV1, and total lung capacity were essentially normal at 81%, 78%, and 81% of predicted, respectively. His DLCO adjusted for alveolar volume was in the normal range at 85% of predicted (Table 1).

Literature review: The mortality from pulmonary disease in patients with DC is reported to be approximately 10-20% [11]. In an effort to better understand the effect of pulmonary disease in patients with DC, we conducted a systematic review of the literature. We searched PubMed through September 1, 2010, using the following terms: "dyskeratosis congenita" combined with each of "interstitial lung disease," "pulmonary fibrosis," or "lung transplantation," as well as "dyskeratosis" combined with "lung." We also identified cases from the bibliographies of those articles, as well as from general reviews of DC. The terms reflective of lung disease used in the clinical reports included "interstitial pneumonitis," "usual interstitial pneumonitis," "pulmonary fibrosis," "restrictive pulmonary function," or "decreased DLCO." To be as comprehensive as possible, our data included reports of patients with DC, as well as those with pulmonary fibrosis who belonged to families where one or more individuals had some features of DC. Data were entered into Microsoft Excel 11.0, (Microsoft, Redmond, WA, USA), and analyzed using Excel and Stata 11.1 (StataCorp, College Station, TX, USA). Analyses included Fisher's exact and Student's t test. The Kaplan-Meier product limit estimator was used to calculate actuarial survival probabilities by age and cumulative incidences in the absence of competing risks; comparisons were made using the Wilcoxon rank-sum test.

We identified 48 DC patients who met criteria for pulmonary disease and 509 patients, reported in the same interval, in whom no pulmonary symptoms were described. Table 2 summarizes the reports of pulmonary complications consistent with or suspicious for pulmonary fibrosis in 37 patients with DC who did not undergo HSCT. Cases 1 - 20 had classic DC with at least two of the three features of DC triad of nail dystrophy, oral leukoplakia and lacy reticular pigmentation. Cases 21 - 37 belonged to families with *TERC *or *TERT *mutations, and had pulmonary fibrosis, with other DC-related features in at least one family member [5,19-22]. In these families, the association of pulmonary fibrosis with DC in the previous generation was made only after a proband in a subsequent generation was identified with clinical features consistent with DC and/or a mutation in a DC gene. For example, in one family (the proband is case 9 in Table 3), the proband's paternal grandmother (case 28 in Table 2) had died from fibrosing alveolitis, and his father (case 29 in Table 2) had received a heart/lung transplant for cryptogenic fibrosing alveolitis [20,23].

**Table 2:** Dyskeratosis congenita (DC) cases with reported pulmonary disease who did not have a hematopoietic stem cell transplant (HSCT)

Case	Sex	DC Triad	Age at Pulmonary Symptoms	Reported Pulmonary Findings	Lung Pathology (Biopsy or Autopsy)	Age Alive	Age Died	References
1	M	3	27	CXR mottled infiltration	Fibrocystic lung dysplasia		27	[29]

2	M	2	23	Respiratory illnesses	Chronic pneumonitis		31	[30]

3	M	3	15	CXR diffuse coarse parenchymal infiltration	Lung segmental fibrosis		17	[31]

4	M	3	11	CXR bilateral hilar enlargement and peripheral markings, bronchopneumonia			11	[32]

5	M	2	12	Interstitial pneumonitis, restrictive lung disease, reduced DLCO	Bronchiocentric granulomatosis, obliteration of respiratory passages	12		[33]

6	F	3	20	Diffuse interstitial pneumonitis, hypoxia, restrictive airway disease	Obliteration of bronchiolar lamina, mild interstitial pneumonitis		20	[33]

7	M	3	39	Mild restrictive impairment, CXR coarse reticular pattern	Pneumothorax, nonspecific collagenous interstitial fibrosis		39	[34]

8	M	2	46	Restrictive impairment, reduced diffusion. CT patchy high density areas and ring-like opacities	Focal fibrosis; UIP	46		[35]

9	M	3	10	Restrictive pulmonary function, CT interstitial thickening and honeycombing	Fibrosis, reticular fibers.		10	[36,37]

10	M	3	30	CT Interstitial lung fibrosis, DLCO 44%	Focal interstitial fibrosis, UIP	38		[38,39]

11	M	2	51	CXR ground glass opacities	Organizing diffuse alveolar damage, UIP		51	[40]

12	M	3	40	Restrictive function, CT ground glass, honeycombing	Fibrosis, UIP		43	[41]

13	F	3	32	Interstitial fibrosis			32.9	[42]

14	M	3	48	Restrictive pattern, DLCO 30%, CT UIP			48	[43]

15	F	3	40	Surgery, XRT, chemotherapy for pharynx SCC at age 28; respiratory insufficiency age 40		40		[44]

16	M	3	37	Pulmonary function, and CT, pulmonary fibrosis			37.7	[45]

17	M	2	41	Pulmonary fibrosis		41		[46]

18	M	3	37	Bronchitis		37		[46]

19	F	3	30	Interstitial fibrosis age 31 yr, left pneumothorax, then right pneumothorax			32	[47]

20	F	2	12	Interstitial pneumonitis		12		[5]

21	F	1	52	Pulmonary fibrosis		52		[5]

22	M	1	68	Pulmonary fibrosis			68	[5,48]

23	M	1	45	Pulmonary fibrosis			47	[5,48]

24	F	0	63	Pulmonary fibrosis			65	[19]

25	M	0	52	Pulmonary fibrosis		52		[19]

26	F	0	54	Pulmonary fibrosis		54		[19]

27	M	0	21	Pulmonary fibrosis		22		[19]

28	F	0	64	Fibrosing alveolitis			64	[20]

29	M	0	60	Cryptogenic fibrosing alveolitis, heart/lung transplant		60		[20]

30	M	0	35	Pulmonary symptoms			35	[21]

31	M	0	24	Pulmonary symptoms			24	[21]

32	M	0	7	Restrictive interstitial pulmonary disease			7	[21]

33	M	0	33	CT lungs fibrotic changes both apical regions		33		[21]

34	F	0	44	Restrictive pattern, decreased diffusion, CT pulmonary fibrosis			46	[22]

35	M	0	32		Diffuse interstitial pulmonary fibrosis		32	[22]

36	M	0	50	Decreased diffusion, CT pulmonary fibrosis			50	[22]

37	F	0	37	Pulmonary fibrosis			37	[22]

All ages are reported in years. The age at pulmonary symptoms is based on the age listed in each report. Triad denotes the classic triad of DC and consists of nail dystrophy, oral leukoplakia, and lacy reticular pigmentation.

Abbreviations: CXR, chest radiograph; XRT, radiotherapy; CT, computerized tomography; DC, dyskeratosis congenita; DLCO, diffusion capacity for carbon monoxide; F, female; M, male; SCC, squamous cell carcinoma; UIP, usual interstitial pneumonia.

**Table 3:** Dyskeratosis congenita (DC) cases with reported pulmonary disease who had a hematopoietic stem cell transplant (HSCT)

Case	Sex	DC Triad	Age at HSCT	Interval from HSCT to Pulmonary Symptoms	Age at Pulmonary Symptoms	Reported Pulmonary Findings	Lung Pathology (biopsy or autopsy)	Age Alive	Age Died	References
1	F	3	11	6	17	6 yr after HSCT restrictive lung disease, DLCO 21%	Interstitial fibrosis		19	[49,50]

2	F	2	4.5	0.1	4.5	Interstitial infiltrates 3 mo after HSCT	Interstitial fibrosis		5	[51]

3	F	3	3	20	23	Respiratory symptoms 20 yr after HSCT	Interstitial fibrosis		23	[50]

4	M	2	26	0.2	26.3	Progressive acute idiopathic pneumonia	Interstitial pneumonitis, pulmonary fibrosis		26	[50]

5	M	2	2	6	8	6 yr after HSCT restrictive airway disease, CT interstitial pneumonitis,	Interstitial fibrosis		10	[52]

6	M	2	5	3	8	3 yr after HSCT CT interstitial pneumonitis	Fibrosis without bronchiolitis	12.5		[53]

7	F	1	7.5	0.5	7.5	Pulmonary complications 6 mo after HSCT			7.5	[54]

8	M	3	16	12	28	Restrictive pattern, DLCO 37%, CT UIP 12 yr after HSCT			28	[43]

9	M	0	27	0.5	27.5	173 days after HSCT, obliterative fibro-alveolitis	Obliterative bronchiolitis		28.3	[20,55]

10	M	2	4		4	Pulmonary disease after HSCT		4		[46]

11	M	3			36	Post-HSCT lung disease			36	[46]

12	M	3	4	7	11	7 yr after HSCT interstitial fibrosis (see Table 1)	Interstitial fibrosis	14		(this report)

The details of the 11 DC cases (plus this case report) who had both pulmonary disease and HSCT are listed in Table 3. All ages are reported in years. Triad denotes the classic triad of DC and consists of nail dystrophy, oral leukoplakia, and lacy reticular pigmentation.

Abbreviations: HSCT, hematopoietic stem cell transplant; CT, computerized tomography; DC, dyskeratosis congenita; DLCO, diffusion capacity for carbon monoxide; F, female; M, male; UIP, usual interstitial pneumonitis.

The reports of pulmonary disease in 12 patients with DC (including our case) who did have a HSCT are summarized in Table 3. The presenting features of pulmonary disease were similar, regardless of whether the patient had received HSCT (Tables 2 and 3). These included persistent dry cough and progressive dyspnea. Evaluations of the patients revealed restrictive lung function impairment, markedly reduced DLCO, patchy or diffuse interstitial infiltrates, and interstitial fibrosis on chest radiographs or CT scans. These findings were documented by lung biopsy or at autopsy in many reported cases.

**Figure 3:**
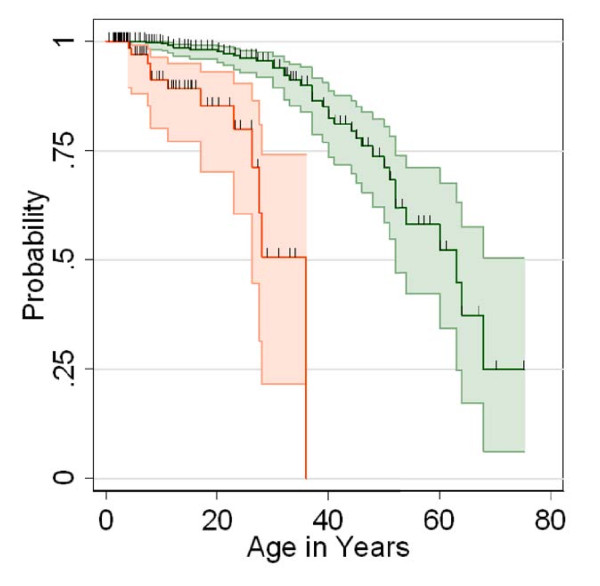
**Age at pulmonary symptoms**. Patients with DC reported in the literature who received a bone marrow transplant (red) had pulmonary symptoms younger than patients who did not have a BMT (green). Data are Kaplan-Meier survival plots, showing cumulative probability of being free of pulmonary symptoms. Shaded areas represent 95% confidence intervals.

We compared the reported clinical features of patients with DC and pulmonary disease based on whether or not they had received an HSCT (Table 4). Pulmonary disease was 2.2-fold more frequent in the HSCT group than in those without HSCT (p = 0.03). Patients who received HSCT developed pulmonary symptoms/disease at an earlier age (median 14 years) than those without HSCT (median 37 years, p < 0.001). In a time-dependent analysis (with censoring at death from non-pulmonary causes), the median survival free of pulmonary disease was 34 years for those who had a HSCT, and 61 years in the untransplanted group (Figure 3, Table 4, p < 0.001). Regardless of HSCT status, once the pulmonary findings became clinically apparent, the pulmonary disease was rapidly progressive in both patient groups. The median survival interval following pulmonary symptoms was brief, 2 years in the untransplanted and 1 year in the transplanted group (p = 0.5). Most patients died at a median interval of 3 months after pulmonary symptoms, range 0 to 8 years (Table 4).

**Table 4:** Comparison of DC Patients with Pulmonary Disease with and without HSCT*

Parameter	HSCT	No HSCT	**P value
Number with pulmonary disease/total number***	12/70	37/488	0.03

Odds Ratio for pulmonary disease *vs*. no pulmonary disease, in patients with HSCT	2.2 (1-4.5)	-	0.03

Age (years) at pulmonary disease in those who developed pulmonary disease, median (range)	14 (4-36)	37 (7-68)	<0.001

Median survival age (years) free of pulmonary disease	34	61	<0.001

Interval (years) from HSCT to pulmonary disease, median (range)	4.5 (0-20)	-	-

Number who died	9/12	24/37	0.8

Interval (years) from pulmonary disease to death in those who developed pulmonary disease, median (range)	0.3 (0-2)	0 (0-8)	0.5

Median survival interval (years) after pulmonary disease,	1	2	0.3

* Data obtained from the cases in Tables 2 and 3.

**P values compare the patients who received HSCT with those who did not receive HSCT.

*** The denominator is the total number of patients with DC who did or did not receive HSCT.

Abbreviations: DC, dyskeratosis congenita; HSCT, hematopoietic stem cell transplantation.

## Discussion

This case report demonstrates that lung transplantation is a viable treatment option for pulmonary fibrosis due to DC. Our report and the literature review suggest that patients with DC who received HSCT are at higher risk of lung-related disease than those who did not receive HSCT. They further illustrate that signs and symptoms of DC develop at different rates in different individuals.

The patient described here received an HSCT for what was thought at the time to be idiopathic SAA. However, in retrospect, the medical records and parental report of toenail dystrophy and geographic tongue (which may have been leukoplakia) suggest that some signs of DC were present but not recognized prior to HSCT. We identified 11 similar cases in the literature: each had received HSCT for SAA and subsequently developed pulmonary disease. In three literature cases plus ours, the HSCT preceded the diagnosis of DC (Table 3; cases 1, 3, 9 and 12). The clinical presentations of the mucocutaneous triad of DC, as well as BMF, pulmonary fibrosis, liver abnormalities, or other related medical problems did not develop at the same rate or in the same order in different individuals. There was variable phenotype expression even between affected individuals within the same family [1,3,22]. Thus, DC may be an under-recognized cause of BMF and IPF.

The evaluation of patients with aplastic anemia typically involves testing for Fanconi anemia and, if that is normal, telomere length testing to rule-out DC [1]. Patients with a severe DC phenotype generally present early with BMF, and are likely to die early or go to HSCT at a younger age. These individuals are also more likely to manifest other DC-related complications, such as pulmonary fibrosis very early in life, as is evident from our current case and our review of published reports. Related disorders of telomere biology have been reported in individuals with apparently isolated IPF who had short telomeres and mutations in *TERT *or *TERC *[24]; these individuals could be considered to have less severe forms of DC. Careful review of the family histories of patients with IPF and a *TERT *or *TERC *mutation may identify individuals with mild manifestations of DC, such as macrocytosis, mild cytopenias, avascular necrosis, cancer, or liver disease [19,20,22,24]. Although they have a similar telomere biology disorder, these individuals may not be as clinically severe as patients with classic DC, and hence may come to medical attention only after they become symptomatic. This likely explains the later age at pulmonary disease in the non-HSCT patients (Table 4, Figure 3). However, since these individuals do have a similar telomere biology disorder, they are likely to be at increased risk of DC-related cancers, such as head and neck squamous cell carcinoma, leukemia and MDS [2].

Our current case and literature review indicate that pulmonary morbidity is one of the major barriers to good quality of life and long-term survival in DC. As is often the case, our literature review was limited by possible reporting bias of more severely affected cases, by inconsistent nomenclature, and by the inability to perform a uniform clinical and pathological review of the cases in the literature. However, even after taking these limitations into account, pulmonary morbidity and mortality remain a major concern in DC; this concern is amplified in patients who have had a successful HSCT. Historically, pulmonary fibrosis may have been under-recognized in patients with DC because of high rates of HSCT-related mortality. The lungs are highly vulnerable to damage by the effects of radiation, chemotherapy, GvHD, and infections related to HSCT. In patients with DC, this pulmonary injury is likely compounded by their underlying disorder. Disease-specific non-myeloablative HSCT regimens in patients with DC have reduced the immediate post-transplant pulmonary or hepatic complications, and have improved short-term survival [25,26]. As more patients with DC survive longer after HSCT, the natural progression of complications of DC, including the development of pulmonary fibrosis, may become a greater clinical challenge, necessitating lung transplant in more patients.

Virtually all lung transplant recipients require three-drug immunosuppressive therapy lifelong because acute and chronic rejection are common complications [27,28]. It is intriguing to note the lack of rejection episodes in our patient despite being on only two-drug immunosuppression. Lack of rejection was observed previously in children who received HSCT for malignancies followed by lung transplantation; this was attributed to an already compromised immunologic state after HSCT in the face of standard post-lung transplantation immunosuppression [18].

## Conclusions

Patients with DC should be monitored with annual or biennial pulmonary function tests including DLCO. The presence of restrictive pattern or moderate to severe reduction in DLCO may suggest pulmonary disease and warrant further studies. Patients with DC who require HSCT should have careful assessment of lung function prior to HSCT. Whenever possible, HSCT preparative regimens should include agents with the smallest potential for pulmonary toxicity. In those with symptomatic pulmonary disease and no evidence of multi-organ failure, irrespective of whether or not they have received HSCT, referral to a lung transplant center should be considered early. As in our case, lung transplantation can provide patients with DC with improved quality of life and a better likelihood of survival.

## Consent

Written informed consent was obtained from the parents of the patient for publication of this case report and any accompanying images. A copy of the written consent is available for review by the Editor-in-Chief of this journal.

## Abbreviations

BMF: bone marrow failure; CT: computerized tomography; CXR: chest radiograph; DC: dyskeratosis congenita; DLCO: diffusion capacity for carbon monoxide; F: female; FEV1: forced expiratory volume in 1 second; FVC: forced vital capacity; GvHD: graft versus host disease; HSCT: hematopoietic stem cell transplant; IPF: idiopathic pulmonary fibrosis; M: male; MDS: myelodysplastic syndrome; PFT: pulmonary function tests; RV: residual volume; SAA: severe aplastic anemia; TLC: total lung capacity; UIP: usual interstitial pneumonia.

## Competing interests

The authors declare that they have no competing interests.

## Authors' contributions

RL and AF were responsible for assessment and management of the patient. CBH performed the lung transplantation. FVW was responsible for pathology review. NG and BPA reviewed the literature. NG wrote the manuscript with the help of BPA and SAS. All authors contributed towards the preparation of this manuscript. All authors read and approved the final manuscript.

## Pre-publication history

The pre-publication history for this paper can be accessed here:

http://www.biomedcentral.com/1471-2326/11/3/prepub
